# Gibberellin mediates daylength-controlled differentiation of vegetative meristems in strawberry (*Fragaria *× *ananassa *Duch)

**DOI:** 10.1186/1471-2229-9-18

**Published:** 2009-02-11

**Authors:** Timo Hytönen, Paula Elomaa, Thomas Moritz, Olavi Junttila

**Affiliations:** 1 Department of Applied Biology, PO Box 27, FI-00014 University of Helsinki, Helsinki, Finland; 2 Viikki Graduate School in Biosciences, PO Box 56, FI-00014 University of Helsinki, Helsinki, Finland; 3 Umeå Plant Science Centre, Department of Forest Genetics and Plant Physiology, Swedish University of Agricultural Sciences, SE 901 83 Umeå, Sweden; 4 Faculty of Science, Department of Biology, Dramsveien 201, University of Tromsø, N-9037 Tromsø, Norway

## Abstract

**Background:**

Differentiation of long and short shoots is an important developmental trait in several species of the Rosaceae family. However, the physiological mechanisms controlling this differentiation are largely unknown. We have studied the role of gibberellin (GA) in regulation of shoot differentiation in strawberry (*Fragaria *× *ananassa *Duch.) cv. Korona. In strawberry, differentiation of axillary buds to runners (long shoot) or to crown branches (short shoot) is promoted by long-day and short-day conditions, respectively. Formation of crown branches is a prerequisite for satisfactory flowering because inflorescences are formed from the apical meristems of the crown.

**Results:**

We found that both prohexadione-calcium and short photoperiod inhibited runner initiation and consequently led to induction of crown branching. In both cases, this correlated with a similar decline in GA_1 _level. Exogenous GA_3 _completely reversed the effect of prohexadione-calcium in a long photoperiod, but was only marginally effective in short-day grown plants. However, transfer of GA_3_-treated plants from short days to long days restored the normal runner formation. This did not occur in plants that were not treated with GA_3_. We also studied GA signalling homeostasis and found that the expression levels of several GA biosynthetic, signalling and target genes were similarly affected by prohexadione-calcium and short photoperiod in runner tips and axillary buds, respectively.

**Conclusion:**

GA is needed for runner initiation in strawberry, and the inhibition of GA biosynthesis leads to the formation of crown branches. Our findings of similar changes in GA levels and in GA signalling homeostasis after prohexadione-calcium and short-day treatments, and photoperiod-dependent responsiveness of the axillary buds to GA indicate that GA plays a role also in the photoperiod-regulated differentiation of axillary buds. We propose that tightly regulated GA activity may control induction of cell division in subapical tissues of axillary buds, being one of the signals determining bud fate.

## Background

Formation of long and short shoots (also called spurs) is a common developmental trait in several economically important species of the Rosaceae family, including apple (*Malus *× *domestica *Borkh.), pear (*Pyrus communis *L.), apricot (*Prunus armeniaca *L.) and sour and sweet cherry (*Prunus ceracus *L., *Prunus avium *L.). Long shoots have long internodes, whereas short shoots are lateral organs with very short internodes and leaf rosette-like appearance. In these species, diverse flowering habits can be found and even the cultivars can differ from each other in this respect. Typically, flowers are borne either on terminal (apple, pear) or lateral (cherry, apricot) buds of short shoots [1-3].

Both shoot types can be found in strawberry (*Fragaria *sp.; e.g. *F*. × *ananassa *Duch., *F. vesca *L.). Strawberry is a perennial rosette plant with short internodes in its crown. Strawberry axillary buds may form both short and long shoots; branch crowns and runners, respectively. Runners are composed of successive units of two long internodes, followed by a leaf rosette (daughter plant). When the terminal bud of the runner forms a daughter plant, the axillary bud in the second node of the runner produces the next sympodial unit. Inflorescences are formed terminally in the short shoots, while the uppermost axillary bud(s) continue vegetative growth of the crown [4,5].

Junebearing strawberry cultivars are facultative short-day (SD) plants; short daylength is the primary factor for induction of flowering, but a strong interaction exists between photoperiod and temperature [6-8]. Daylength also controls vegetative differentiation of axillary buds in strawberry. In long-day (LD) conditions, runners are formed, and in SD crown branching occurs [4,9,10]. Crown branching is a prerequisite for satisfactory flowering, and consequently, for berry crop production because the inflorescences are formed from the apical meristems of the crown and axillary buds containing at least two leaf initials [10,11].

In the late 1950s, Guttridge [12] proposed that a transmissible hormone produced in LD enhances petiole elongation and runnering and inhibits flowering in strawberry, the effects mimicked by gibberellin (GA) application [13-16]. Inhibitors of GA biosynthesis have the opposite effect on vegetative growth [17-20], but their effect on flowering time is unknown. Prohexadione-calcium (Pro-Ca), the inhibitor of GA 3-oxidase enzyme [21], has recently been shown to effectively inhibit runner formation and enhance crown branching in strawberry under field conditions [22], with a concomitant increase in berry yield [23].

In strawberry, the early 13-hydroxylation pathway appears to be a predominant GA biosynthesis pathway [24-26]. In this pathway, GA_12 _is 13-hydroxylated to GA_53_, which is further converted to other GAs in cytosol by three enzymes, encoded by small gene families [27]. GA 20-oxidases are multifunctional enzymes responsible for conversion of GA_53 _to GA_20 _through GA_44 _and GA_19_. GA 3-oxidases produce bioactive GA_1 _from GA_20_, and GA 2-oxidases catalyse the inactivation steps GA_20 _to GA_29 _and GA_1 _to GA_8_, respectively. Many of the GA oxidase genes are subjected to tight developmental [28,29] and/or light (light quality, photoperiod) regulation [30-32].

GA receptors have recently been cloned, one from rice and three, *GID1a*, *GID1b *and *GID1c*, from *Arabidopsis *[33,34]. Binding of GA to GID1 leads to a physical interaction between GID1 and DELLA transcriptional repressors [35], the central components of the GA signalling pathway that suppress GA-mediated growth responses [36]. SLY1 F-box protein, a component of E3 ubiquitin ligase SCF complex (SKP/Cullin/F-box), recruits the receptor-bound DELLA proteins and targets them to degradation in 26S proteasome, leading to the release of plant growth from DELLA-mediated restraint [37].

The GA pathway is tightly regulated by GA itself at multiple levels, a phenomenon called GA signalling homeostasis [38]. Some of the GA biosynthetic (*GA20ox *and *GA3ox*) genes and positive regulators of GA signalling (*GID1*, *SLY1*) are feedback-regulated, and catabolic genes (*GA2ox*) and negative signalling components (*DELLA *genes) are feed-forward regulated by GA. In addition, a reduced level of one DELLA protein can be compensated by accumulation of another DELLA [35].

Despite the importance of short and long shoot differentiation in many cultivated species of the Rosaceae family, the physiological and genetic mechanisms of this differentiation remain largely unknown. In this paper, we studied the role of GA in regulating short/long shoot differentiation in strawberry, which is one of the best-developed model species for Rosaceae [39]. We report the effects of daylength and GA biosynthesis inhibitor Pro-Ca on axillary bud differentiation, GA levels and GA signalling homeostasis. The results indicate that GA is one of the key signals affecting axillary bud differentiation.

## Results

### Photoperiod, prohexadione-calcium and GA_3 _affect the differentiation of strawberry axillary buds

**Figure 1:**
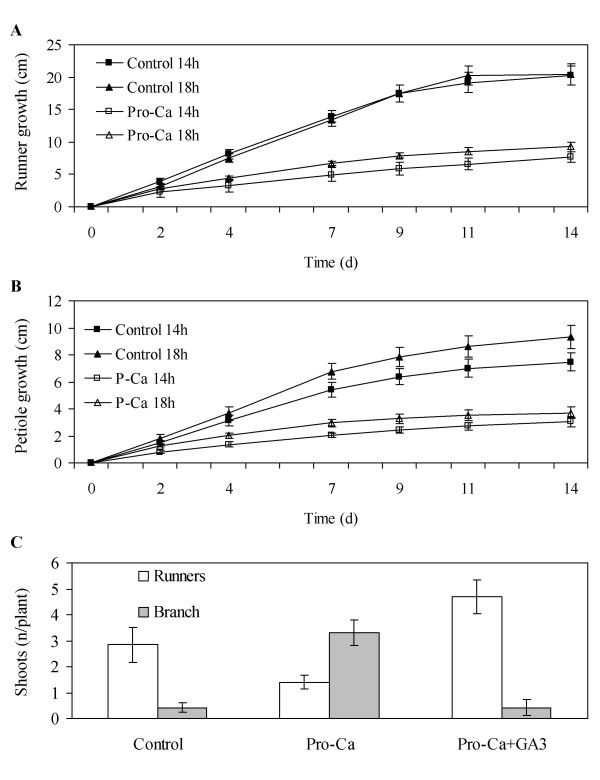
**Effect of prohexadione-calcium (Pro-Ca), GA_3_, and photoperiod on strawberry vegetative growth**. The elongation growth of runners (**a**) and petioles (**b**), respectively. LD-grown plants were treated with 50 mg l^-1 ^Pro-Ca or with water (control) and moved to daylengths of 14 or 18 h (n = 10). The youngest petiole and runner were marked at the beginning of the treatments, and their lengths were measured three times per week. Petiole growth was measured until the termination of elongation and runner growth until the formation of the first daughter plant. **c**: Effect of Pro-Ca and GA_3 _on the number of branch crowns and runners in LD (18 h). The foliar application of 50 mg l^-1 ^Pro-Ca or water (control) was performed at the beginning of the experiment. For half of the plants, 25 μg of GA_3 _in 5 μl of 70% ethanol was applied twice, one and two weeks after Pro-Ca treatment, to the tip of the youngest leaf, and control plants were treated similarly with 70% ethanol. The number of branch crowns and runners were counted 10 weeks after the ProCa/water treatments (mean of 10 plants ± SE).

As shown in Table 1, 10-, and 14-h photoperiods (SD) promoted crown branching equally, whereas no branch crowns were found in plants grown under an 18-h photoperiod (LD). Pro-Ca treatment of 50 mg l^-1 ^reduced the elongation of runners and petioles rapidly in all photoperiods (Figure 1A, B). This treatment also strongly enhanced crown branching and reduced the number of runners compared with control plants (Figure 1C). The effect of Pro-Ca was completely reversed by 25 μg of GA_3 _(*p *< 0,001) applied twice to the youngest fully open leaf, suggesting that the reduced level of active GA was responsible for the altered bud fate. Our data show that both SD and Pro-Ca treatment similarly promoted branch crown formation, whereas GA_3 _or LD enhanced runner formation from axillary buds of the strawberry crown.

**Table 1:** Effect of photoperiod (h) on strawberry vegetative growth.

Photop.	1^st ^petiole	2^nd ^petiole	Runner	Nodes	Crown branches
10	11.6 ± 0.5b^z^	8.6 ± 0.4a	37.7 ± 3.8b	2.9 ± 0.3b	1.7 ± 0.4
14	13.6 ± 0.5ab	12.3 ± 0.2b	39.7 ± 2.6b	2.9 ± 0.3b	2.0 ± 0.8
18	14.7 ± 0.6a	15.4 ± 0.8c	120.4 ± 3.0a	8.5 ± 0.3a	0.0

^z^Different letters denote a statistically significant difference (*p *≤ 0.05).

The final length (cm) of the longest runner and the first and second petiole formed after the movement of plants from LD (18 h) to indicated daylength treatments. The number of nodes per longest runner and the number of crown branches per plant were counted 10 weeks after the beginning of the daylength treatments. Values are averages of ten plants ± SE.

### Short photoperiod promotes crown bud differentiation on strawberry runners

**Figure 2:**
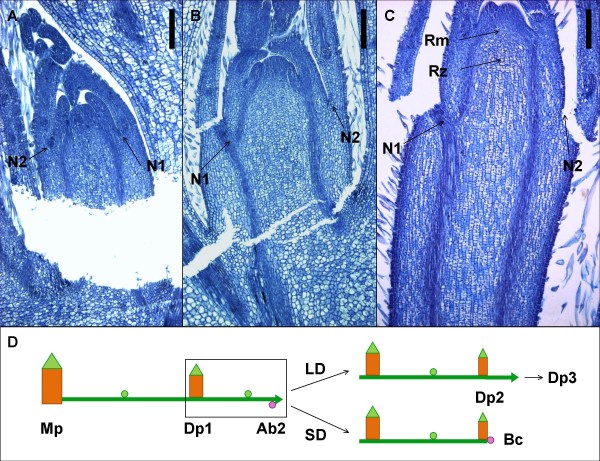
**Photoperiodic regulation of axillary bud differentiation in strawberry**. Longitudinal sections of non-differentiated (**a**) and differentiated (**b **and **c**) axillary buds. Buds were collected from SD-grown 6-day-old runner tip (a) and SD- or LD-grown 10-day-old runners (b and c, respectively). **d**: Schematic drawing showing the growth habit of the strawberry runner. Under LD conditions, the axillary bud of the mother plant (Mp) produces a runner, which consists of units of two long internodes and a daughter plant (Dp 1, 2, 3). When the terminal bud of the runner produces a new Dp, the second axillary bud of the runner (Ab2; axillary bud #2) forms the next sympodial unit, continuing the growth of the runner in LD. In SD, runner growth ceases and Ab2 forms a branch crown (Bc). N1, N2 = node 1 or 2; Rm = rib meristem, Rz = rib zone. Scale bar = 200 μm.

Based on our observations on axillary bud differentiation in crowns, we wanted to test whether the developmental fate of axillary buds on runners is similarly regulated by photoperiod. These buds are more accessible and easier to collect than buds of the crown and could provide a good model system for analytical and molecular studies. Strawberry runners consist of sympodial units of two long internodes, followed by a daughter plant with short internodes (Figure 2D). We tested photoperiodic responses of the axillary bud on the second node of the runner (axillary bud #2). In an 18-h photoperiod, axillary bud #2 formed a new sympodial unit after the production of a daughter plant by the apical meristem of the previous unit. This was continuously repeated until the end of the experiment, when the longest runners consisted of about four sympodial units, i.e. eight internodes (Table 1). In contrast, both 10- and 14-h photoperiods (SD) stopped runner growth after the formation of the first or second daughter plant. In this case, axillary bud #2 differentiated into a crown bud instead of continuing runner growth.

To further study the photoperiodic control of bud differentiation, newly emerged 2- to 3-cm-long (~2-day-old) runners were marked 4, 8, 12 and 16 days after transfer to SD. Axillary bud #2 differentiated into a normal branch crown in all runners (n = 10) marked 8 days or later after transfer to SD. In runners marked after four days in SD, the differentiation of axillary bud #2 into crown buds was incomplete; four out of nine buds formed one elongated internode (> 3 mm) before producing a normal leaf rosette. Thus, axillary bud #2 differentiated into a crown bud in runners, which started to form 2 – 6 days after transfer to SD. In conclusion, the differentiation of runner axillary bud #2 can be strictly controlled, and therefore, it provides a good model to study bud differentiation in strawberry.

### Timing of differentiation of axillary buds in runners

Anatomical studies of developing axillary buds on 6- and 10-day-old runners show that differentiation to either crown or runner bud takes place between these time points. On day six, SD- and LD-grown axillary buds were morphologically almost indistinguishable (Figure 2A), except that the number of cells in the first (oldest) internode was slightly higher in LD than in SD. In 10-day-old runners the buds developed in SD and LD were clearly different (Figure 2B, C). In SD buds, probably already determined as crown buds, the cell length had not increased, and the number of cells in the longitudinal axis of the first internode of the bud was almost the same as four days earlier; 26 and 30 cells (n = 5) in 6- and 10-day-old runners, respectively. By contrast, in LD, the cell number had increased, and the length of LD buds was double that of SD buds in 10-day-old runners (Figure 2C). In addition, clear longitudinal cell files were found in late LD buds compared with less regular organization of cells in SD (Figure 2B, C).

We wanted to confirm the results for timing of differentiation with Pro-Ca treatments. Nineteen 4- to 8-day-old runners were treated with 200 mg l^-1 ^Pro-Ca under LD conditions, and the developmental fate of axillary bud #2 was determined. Axillary buds of all 4- to 6-day-old runners (8 runners) treated with Pro-Ca differentiated into branch crowns, whereas the buds in older runners (11 runners) were only partially affected; the branch crowns with an elongated first internode (5 – 10 mm) or short runners were formed. In runners older than eight days, axillary bud #2 was already committed to developing into a runner and Pro-Ca treatment could not alter or restore its fate. Thus, under the LD conditions applied, the identity of axillary bud #2 is determined in 'Korona', when the age of the runner is between 6 and 8 days.

### Pro-Ca treatment and short photoperiod lead to reduced GA levels

**Figure 3:**
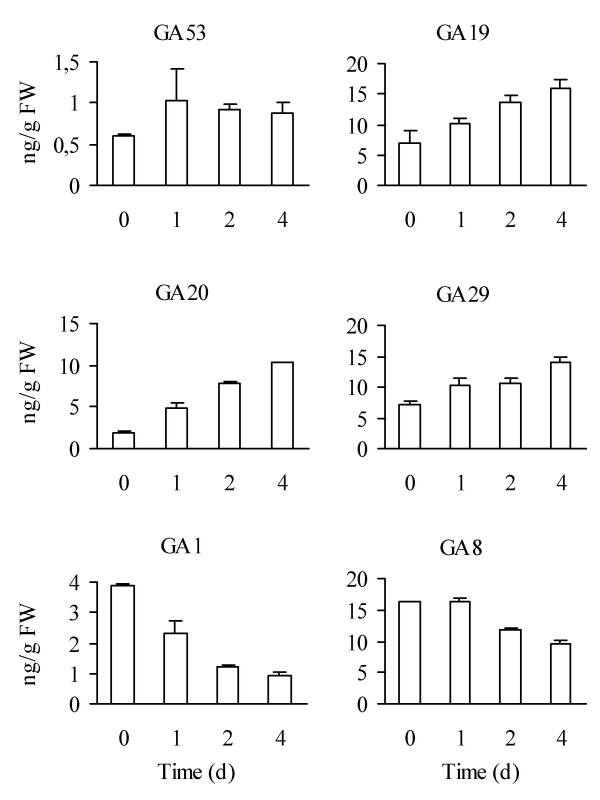
**Effect of prohexadione-calcium (Pro-Ca) on gibberellin content of strawberry runner tips**. Duplicate samples containing 5 – 6 runner tips were collected before the Pro-Ca treatment and at 1, 2 and 4 days after treatment. All samples were collected from LD grown plants during the linear growth stage of the runner.

Pro-Ca treatment of whole plants resulted in rapid changes in the levels of several 13-hydroxylated GAs in runner tips (Figure 3). The level of active GA_1 _was nearly halved already one day after the Pro-Ca treatment and three days later only one quarter was left compared with non-treated control plants. In contrast, after four days of treatment, the levels of GA_20 _and GA_19_, the immediate precursors of GA_1_, were increased five- and twofold, respectively. Changes in GA_20 _and GA_1 _levels were reflected in the levels of their 2β-hydroxylation products, GA_29 _and GA_8_, respectively.

**Figure 4:**
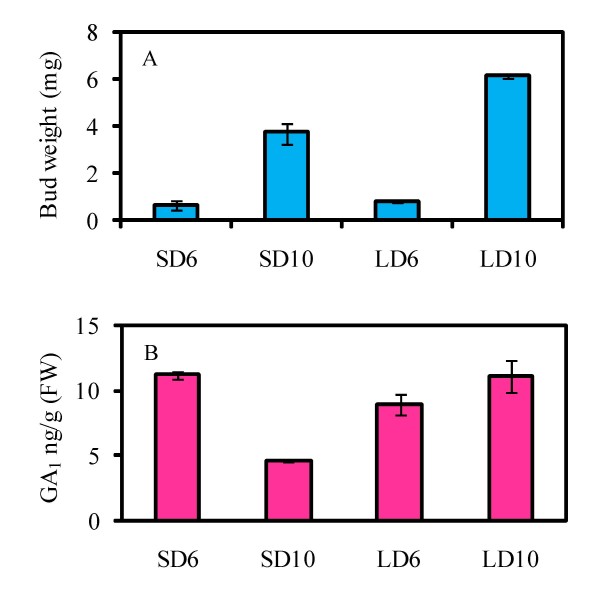
**Effect of daylength on GA_1 _concentration of strawberry axillary buds**. The bud weight (**a**) and GA_1 _concentration (**b**) of non-differentiated and differentiated axillary buds collected from 6- or 10-day-old runners (SD/LD6 and SD/LD10, respectively) of plants grown 12 – 18 days under SD or LD conditions. Values are means ± SE of three independent bud samples containing 7 – 10 (SD/LD6) or 3 – 4 (SD/LD10) buds.

The analysed samples consisted of both bud meristems and internode tissues. To obtain more precise information about GAs in the buds themselves, we collected axillary bud samples from runners of SD- and LD-grown strawberry plants and analysed the levels of bioactive GA and the expression levels of several candidate genes. The first samples consisted of buds just prior to the determination of bud identity (axillary bud #2 from a 6-day-old runner, Figure 2A), and the next sampling was done four days later, when bud differentiation was clearly visible (Figure 2B, C). We observed that GA_1 _level was slightly higher in SD-grown axillary buds than in LD buds prior to determination of bud identity (Figure 4B). However, four days later, an over 50% reduction in GA_1 _level occurred in SD buds, whereas in LD-grown buds the GA_1 _level remained constant.

### Short photoperiod reduces GA sensitivity of axillary buds

If reduced level of active GA found in SD is responsible for changes in bud fate, GA treatment should restore runner initiation from axillary buds under SD. Therefore, we applied 25 μg of GA_3 _to the SD-grown 2- to 4-day-old runner tips and observed the final length of the treated runners (two internodes preceding axillary bud #2) and the fate of axillary bud #2. This GA treatment did not significantly increase the length of the runners in SD, and the fate of axillary bud #2 was affected only in a few plants (Table 2). Three out of nine buds initiated runner after GA treatment in SD, and all newly formed runners remained short (1 – 4 cm). However, moving of plants to LD after GA treatment had a strong effect on the length of treated runner and fully restored the runner growth from axillary bud #2. LD without GA treatment, in contrast, neither increased the final length of the runners nor affected the fate of axillary bud #2. These findings suggest that, in addition to bud GA_1 _levels, photoperiod affects the sensitivity of buds and elongating runners to GA. Similarly, the growth of strawberry petioles in SD was reduced before the GA_1 _levels were reduced [25].

**Table 2:** Effect of photoperiod on the GA responsiveness of strawberry runners and axillary buds.

	SD-GA_3_	SD to LD-GA_3_	SD +GA_3_	SD to LD +GA_3_
Runners	0	1	3	13
Branch crowns	10	12	6	0
Runner length (mm)	129 ± 47	142 ± 44	145 ± 48	247 ± 35

Plants were grown in SD for 10–18 days, and 25 μg of GA_3 _(in 70% EtOH) was applied to the tip of 2- to 4-day-old runners, after which the plants were either left in SD or moved to LD conditions. Control plants were treated with EtOH only. The fate of axillary buds was evaluated, and the final length of the treated runner (mm, two internodes preceding the axillary bud) was measured after 28 days.

### Identification of putative GA biosynthesis, signalling and target genes

For gene expression studies, we isolated full-length cDNAs encoding putative strawberry GA 20-oxidase (*FaGA20ox*) [GenBank: DQ195504] and GA 3-oxidase (*FaGA3ox*) [GenBank DQ195505]. The EST clone for a putative GA 2-oxidase was sequenced earlier [40]. A search on public databases revealed no additional GA oxidase sequences in strawberry. In addition to GA biosynthetic genes, we cloned a 816-bp fragment of putative GA signalling repressor, *FaRGA *[GenBank: DQ195503], and identified homologues of putative GA signalling genes *SLY1 *and *GAI *as well as two putative GA receptors, *GID1b *and *GID1c*, from public databases. In addition, a 558-bp fragment of a putative *SPY *homologue was found from our own *Fragaria vesca *EST collection (Mouhu K et al., unpublished data). As putative target genes for GA regulation we searched strawberry sequences homologous to *Arabidopsis *GA regulated genes and identified *XERICO *[41] and *FaGAST *[42]. XERICO is a RING-H2 zinc finger protein, and FaGAST is homologous to the GA-induced GASA family of *Arabidopsis *and the GAST family of tomato [42]. For the sake of clarity, common names of *Arabidopsis *genes are used for corresponding strawberry EST sequences.

### Effect of Pro-Ca on gene expression in runner tips

Several genes, involved in GA biosynthesis and signalling, are regulated by GA in *Arabidopsis *[35,41]. We compared the expression levels of ten putative GA biosynthesis, signalling and target genes in Pro-Ca-treated and non-treated strawberry runner samples two and four days after treatment. At these time points, bioactive GA_1 _levels were reduced by 68% and 74%, respectively (Figure 3). The reduced GA_1 _levels were associated with upregulation of GA-biosynthetic gene *FaGA3ox *and severalfold downregulation of *FaGA2ox *(Table 3). Based on RNA-blot analysis, Pro-Ca did not affect *GA20ox *expression levels (data not shown).

**Table 3:** Effect of Pro-Ca and photoperiod on the expression of putative GA biosynthesis, signalling and target genes in strawberry.

	Fold change			
	
	P2 vs. C2	P4 vs. C4	SD6 vs. LD6	SD10 vs. LD10
GA_1 _conc.	-68%	-76%	+26%	-58%

	*GA biosynthetic genes*			
*GA3ox*	1.744	2.099	0.933	0.476
*GA2ox*	0.236	0.170	0.601	0.140

	*GA signalling genes*			
*GID1b*	2.379	3.158	2.953	2.038
*GID1c*	1.107	1.140	1.400	0.556
*GAI*	0.544	0.490	0.799	0.568
*RGA*	0.669	0.651	1.093	0.611
*SLY*	1.591	2.216	1.769	1.677
*SPY*	0.770	0.753	1.270	0.852

	*GA target genes*			
*GAST*	1.238	2.039	1.771	4.206
*XERICO*	1.649	4.480	3.055	1.568

Fold-change values of Pro-Ca-treated vs. water-treated (control) runner tips at 2 (P2/C2) and 4 days (P4/C4) after treatment, and SD- vs. LD-grown axillary buds before (SD/LD6) and after (SD/LD10) the determination of bud fate are shown. *UBI14 *or *UBI14 *+ *Rubisco small subunit 1a *were used as control genes in bud samples and runner tip samples, respectively. Values are means of two biological and three technical replicates.

Positive regulators of GA signalling, *SLY *and *GID1b*, were upregulated in Pro-Ca-treated plants, as was the *XERICO *homologue (Table 3). Among the negative regulators of GA signalling, both DELLA genes (*GAI *and *RGA*) were slightly downregulated by Pro-Ca. In contrast to earlier studies [42], *GAST *was upregulated in samples with reduced GA_1 _levels four days after Pro-Ca treatment.

### Effect of photoperiod on gene expression in axillary buds

In addition to runner tips, we analysed gene expression in axillary bud #2. GA oxidase genes *GA3ox *and *GA2ox *were twofold and sevenfold reduced, respectively, in differentiated SD grown buds compared with LD buds (Table 3). In contrast, no clear differences between SD and LD were found in non-differentiated buds. Reduced *GA3ox *mRNA abundance in SD buds was contrary to our results for Pro-Ca-treated runners, indicating that photoperiod rather than feedback mechanism controls transcription of this gene in buds. Therefore, we analysed *GA3ox *expression levels also in runner tips grown in SD and LD conditions for 8 or 12 days, but no significant differences were found in these samples. However, *GA2ox *was 7.4-fold downregulated in SD-grown axillary buds compared with runner tips, indicating more active GA signalling in runners. In conclusion, *GA3ox *and *GA2ox *expression levels were unaffected by photoperiod in elongating runner tissues, but were downregulated in SD-grown axillary buds with crown bud identity.

Based on similar reductions in GA_1 _levels in SD-grown buds and Pro-Ca-treated runners, we expected to observe upregulation of *GID1b, SLY*, *XERICO *and *GAST *and downregulation of both DELLA genes in SD-grown differentiated axillary buds. This was in fact true for all of these genes (Table 3). However, the transcription of *XERICO*, *GID1b*, *SLY *and *GAST *was clearly upregulated also in non-differentiated SD-grown buds, although the GA_1 _level was not reduced (Table 3), possibly indicating some early reduced GA signalling event.

## Discussion

Our results confirm earlier studies on photoperiodic control of vegetative growth in strawberry and provide further evidence for involvement of GA in regulation of axillary bud differentiation into branch crowns or runners in strawberry cv. Korona. Differentiation of axillary buds in the strawberry crown is strictly regulated by photoperiod [9,10]. We have shown that this also applies to axillary buds of runners (axillary bud #2), which can be used as models to study the regulation of bud fate. The critical photoperiod to induce runner formation in 'Korona' was between 14 and 18 h in axillary buds of both crowns and runners. Moreover, these buds responded similarly to Pro-Ca and GA_3 _applications, indicating a similar regulation of bud fate in crown and runners.

Different GA biosynthesis inhibitors have been shown to affect strawberry vegetative development, including runner formation and crown branching [17,18,22]. We found that Pro-Ca rapidly reduced elongation growth of petioles and runners and inhibited runner initiation from axillary buds after 1 – 2 days. The inhibition correlated with a moderate (41 – 68%) reduction in the level of GA_1_, which has been shown to be the predominant bioactive GA in strawberry [this study, [24-26]]. Interestingly, a similar reduction in GA_1 _level was found in SD-grown differentiated axillary bud #2 compared with differentiated LD buds, while GA_1 _levels were not clearly affected by photoperiod in four days younger non-differentiated buds. Olsen *et al. *[43] reported a similar drop in GA_1 _level in subapical tissues of *Salix pentandra *before the cessation of elongation growth.

GA_3 _application completely reversed the effect of Pro-Ca on axillary buds, but was only marginally effective on SD-grown axillary bud #2. However, the movement of plants to LD after GA_3 _treatment fully restored runner formation from this bud, whereas the movement of non-treated plants to LD did not affect bud fate. According to these data, a moderate drop in GA_1 _level in SD-grown axillary buds and in Pro-Ca-treated plants may alter bud fate, but it is also possible that photoperiod alters GA responsiveness of the buds. A similar effect has been observed in *Salix pentandra *[44].

The first anatomical sign of runner identity in LD-grown axillary buds is rapid longitudinal cell division occurring in the first internode. In contrast to LD-grown buds, cell division in internodes of SD buds is more disorganized and ceases soon after determination of branch crown identity. GA has been shown to affect elongation growth both by controlling alignment of cortical microtubules [45,46] and by controlling cell division [47-49]. Accordingly, higher GA level/action in LD-grown strawberry axillary buds, compared with SD buds, may induce the transverse orientation of microtubules in the longitudinal axis of the cells, thereby determining the direction of cell division. Furthermore, GA may be a major inductive signal starting cell division in strawberry LD buds.

Gene expression analysis revealed that the expression of *GA2ox*, involved in GA inactivation, was sevenfold downregulated after both Pro-Ca and SD treatment and correlated with reduced GA_1 _levels. This suggests a strong feed-forward regulation, which is a common feature for several GA biosynthetic genes [35,38,41]. *GA3ox *expression was upregulated in Pro-Ca-treated plants, also indicating feedback regulation. However, we did not observe similar feedback control in SD-grown axillary buds. Instead, *GA3ox *expression was reduced in relation to changed GA_1 _level, suggesting that photoperiod may regulate its activity. Photoperiodic regulation of *GA3ox *was further tested in SD- and LD-grown runner tips, but no changes were found after 8 or 12 SD cycles compared with LD. We therefore postulate that *GA3ox *in strawberry is not necessarily regulated by photoperiod or that its regulation in buds and in runner tips is different. A local, bud-specific decrease in GA activity is supported by the finding that *GA2ox *was sevenfold downregulated in SD-grown axillary bud #2 compared with runner tips, while under LD it had a similar expression level in both tissues. These data are consistent with earlier findings that the biosynthesis of active GA occurs at the site of GA action [43,50-52]. In strawberry axillary buds, subapical tissue is a potential site of GA biosynthesis and action. Thus, some unknown photoperiod-regulated factor may activate or repress runner initiation by affecting the biosynthesis of GA_1 _in the subapical tissues of the bud, consequently determining bud fate.

In addition to GA biosynthetic genes, we also identified six putative GA signalling and two putative target genes in strawberry. Most of these transcripts showed similar GA regulation in strawberry as the corresponding genes in *Arabidopsis *[35,41]. The expression of putative positive regulators of GA signalling, *GID1b *and *SLY*, was upregulated, while that of negative regulators, *RGA *and *GAI*, was downregulated by Pro-Ca and SD, following changes at the level of GA_1_. Moreover, GA-responsive *XERICO *and *GAST *were upregulated after both Pro-Ca and SD treatments. The GA and SD regulation of these genes, in addition to *GA2ox*, indicates strictly regulated GA signalling homeostasis and reflects more active GA signalling in LD, providing molecular evidence for GA regulation of strawberry axillary bud differentiation.

*XERICO*, *GID1b*, *SLY *and *GAST *were upregulated also in non-differentiated SD-grown buds, although the GA_1 _level was unchanged. This may be explained by GA-independent regulation of these genes or by an early event of altered responsiveness of buds to GA in SD. This is supported by the finding that the response of strawberry axillary buds and runners to exogenous GA_3 _was indeed reduced in SD. In *Arabidopsis*, the overexpression of XERICO, a GA-downregulated RING-H2 zinc finger protein, increases ABA accumulation, being a possible link between antagonistic ABA and GA pathways [41]. Moreover, ABA, induced by flooding, has been shown to regulate responsiveness to GA in deepwater rice intercalary meristems [53]. Thus, threefold upregulation of *XERICO *in non-differentiated axillary buds in SD compared with LD could indicate that *XERICO *and ABA are regulators of axillary bud fate upstream of GA.

One interesting feature of strawberry is an obvious antagonism between runner formation and flowering induction. These processes have opposite responses to both exogenous GA and photoperiod, thereby raising the question about common regulation [5,9,13]. However, in *Fragaria vesca *L., seasonal flowering (SD genotype) vs. perpentual flowering as well as runnering vs. non-runnering phenotypes are determined by different single genes, "seasonal flowering locus" and "runnering locus", seasonal flowering and runnering being dominant characters [54,55]. Emerging data from different plant species indicate that CONSTANS (CO) and FLOWERING LOCUS T (FT) are central regulators of daylength-dependent flowering induction and that FT protein acts as a long-distance signal [56-58]. Interestingly, the CO/FT module may have a more general role in daylength responses, including growth cessation and tuberization [59-61]. Hybrid poplar *CENTRORADIALIS-LIKE 1 (CENL1)*, a gene belonging to same gene family as *FT*, also coincides with growth cessation [62]. This gene is expressed mainly in the rib meristem area, and is downregulated in the shoot apex by SD. A gene homologous to *CENL1 *is also found in strawberry, and our gene expression data show that it is upregulated threefold in differentiated LD-grown axillary buds compared with SD buds. Moreover, anti-sense expression of apple *TFL1 *homologue in apple reduces the juvenile phase and produces dwarf plants, supporting the role of TFL1 in the regulation of stem elongation [63]. The role of the CO/FT module and TFL1 in photoperiodic responses in strawberry warrants further study. In contrast to other plants, studies in *Lolium temulentum *indicate that photoperiodic flowering is mediated by GA_5 _[64]. However, the link between photoperiodic effects and GA biosynthesis/action also remains an open question [65].

## Conclusion

Our results indicate that GA functions as one of the key signals regulating the developmental fate of vegetative buds in strawberry. Some photoperiod-dependent factor(s) regulates GA biosynthesis and/or signalling in the subapical tissues of strawberry axillary buds, thereby determining bud fate. In light of these data, identification and functional characterization of the *Fragaria *"runnering locus" gene is of great interest. Strawberry is a potential model plant for molecular studies of short shoot/long shoot differentiation, and these studies may uncover new possibilities for controlling yield formation in various small fruit and fruit species.

## Methods

### Plant material and growing conditions

Cultivated strawberry (*Fragaria *× *ananassa*, Duch.) cv. Korona plants were used for the experiments carried out in greenhouse conditions at the University of Helsinki. Plants were propagated from runner cuttings, which were rooted on sphagnum peat. Plants were irrigated when needed with a complete fertilizer solution containing Ca(NO_3_)_2 _(Kemira GrowHow, Finland) and a strawberry fertilizer mixture (Mansikan täyslannos, Kemira GrowHow, Finland) in a ratio of 1:3. Air temperature in the greenhouse was maintained at 18/15°C (day/night), and daylight was supplemented with high pressure sodium (HPS) lamps (Osram NAV-T 400W, 90 ± 10 μmol m^-2 ^s^-1^) for 18 h daily.

### Daylength treatments

All short-day (SD) treatments (photoperiod 10, 12 or 14 h) were carried out at the greenhouse using darkening curtains, while long-day (LD) control treatments (photoperiod 18 h) were conducted in a similar greenhouse compartment without curtains. The main light period (the shortest photoperiod in each experiment) was given during natural day, which was 7 – 12 h during the experiments (October – March). During the main light period plants were illuminated by HPS lamps (90 ± 10 μmol m^-2 ^s^-1 ^at plant height plus natural light), and incandescent lamps were used for low-intensity daylength extension (5 ± 1 μmol m^-2 ^s^-1 ^at plant height), yielding nearly equal total irradiance in all photoperiods. Air temperature was 18°C during the main light period, otherwise 15°C.

### Pro-Ca and GA_3 _treatments

Pro-Ca 10% w/w a.i. (BAS 125 growth regulator, BASF, Ludwigshafen, Germany) was dissolved in water and diluted to indicated concentrations. Two to three drops of Tween 20 was added to facilitate uptake of Pro-Ca. Water containing Tween 20 was applied to control plants. Pro-Ca sprays were applied to drip-point, using a hand sprayer. To examine whether Pro-Ca-induced growth changes were caused by decreased GA levels, 25 μg of GA_3 _(Duchefa, Haarlem, The Netherlands) in 5 μl of 70% ethanol was applied to the tip of the youngest fully open leaf twice, one and two weeks after Pro-Ca treatments. Control plants were treated similarly with ethanol containing no GA_3_. Runner GA treatments were carried out by applying 25 μg of GA_3 _in 5 μl of 70% ethanol to the runner tip once, and control plants were similarly treated with 70% ethanol.

### Gibberellin extraction, purification and detection

All samples for GA analysis were collected 5 – 7 h after the beginning of the subjective day to avoid possible diurnal changes. Runner tip samples 3 cm in length were collected from Pro-Ca-treated (50 mg l^-1 ^a.i.) plants 1, 2 and 4 days after treatment and from water-treated control plants (day 0). Samples were collected during the linear growth stage of the runners, freeze-dried and stored at -80°C until GA extraction. Runner tip samples of 1 – 2 g (FW) were homogenized, and GAs were extracted with 85% aqueous methanol at 4°C in a shaker with added internal standards, 10 ng (runner tips) each of [^2^H_2_] GA 1, 4, 8, 9, 19, 20, 29, 53 and [^2^H_1_] GA 44 (all deuterated GAs were obtained from Prof. L. Mander, Australian National University, Canberra). Purification of GAs was done according to Junttila *et al. *[66], with the following modifications: Volumes were scaled down and purification was performed in 2 ml Eppendorf tubes. After partitioning of samples against ethylacetate, borax/hydrochloric acid pH 8 buffer (Einecs, Fluka) was applied to the samples to facilitate pH adjustment. Bead volume of Sephadex columns was 0.5 ml. In addition, smaller (100 mg) C_18_- and aminopropylsilyl solid-phase extraction columns (Sep-Pak, Waters, Milford, MA, USA) were used. HPLC purification and fractionation of samples and GA analysis by GC-MS were done at the University of Tromsø, as described earlier [66]. The instrumentation consisted of a PAL autoinjector (CTC Analytics AG, Zwingen, Switzerland) and a Factor Four capillary column (VF-5 ms, 30 m × 0.25 mm ID; 0.10 μm film thickness, Varian, Middelburg, The Netherlands) installed in a Trace GC connected to a Trace DSQ single quadropole mass selective detector (ThermoFinnigan, Austin, TX, USA). XCalibur 1.3 software (ThermoFinnigan) was used to control the system and to review the data. For each GA and its ^2^H analogue, at least two characteristic ions were recorded. Two biological duplicates were performed for each sample.

Axillary bud samples of 5 – 15 mg (FW) were collected from LD (18 h) and SD (12 h) grown 6- and 10-day-old runner tips under a dissecting microscope, frozen in liquid nitrogen and stored at -80°C. Homogenized bud samples were extracted with added internal standards (100 pg each) and GAs were purified according to King et al. [64]. GA analyses by GC-MS were done at the Umeå Plant Science Centre, as described earlier [64].

### RNA extraction, cDNA synthesis and gene cloning

Strawberry total RNA was isolated using the pine tree method [67]. mRNA was extracted with a NucleoTrap mRNA purification kit (BD Biosciences, Palo Alto, NC, USA). cDNA synthesis was performed using the 1^st ^strand cDNA synthesis kit (Roche Diagnostics, Indianapolis, IN, US). cDNA fragments of *FaGA20ox*, *FaGA3ox *and *FaRGA *genes were amplified from runner tip cDNA samples by a standard RT-PCR protocol using Taq Polymerase (Roche Diagnostics). The following degenerate primers were used: *FaGA20ox*; 5'-TGG CCI GAY SAB GAR AAR CC-3' (sense) and 5'-CCR TTY GAW AGI GCC ATR AA-3' (anti-sense), *FaGA3ox*; 5'-AAR CKY ATG TGG TIY GAR GGI TT-3' (sense) and 5'-TCB GTR TGI GCD SCI AGM CCC AT-3' (anti-sense), *FaRGA*; 5'-GCW CAR AAR CTY GAR CAG CTT GA-3' (sense) and 5'-AAV GCT TCV AGR ATV GCT TGA TT-3' (anti-sense). The BD Smart RACE cDNA amplification kit (BD Biosciences) was used to amplify the 5' and 3' ends of *FaGA20ox *and *FaGA3ox *genes. All PCR products were cloned into a pGEM T Easy vector (Promega, Madison, WI, USA) and sequenced by ABI Prism 3100 16-capillary sequencer (Applied Biosystems, Foster City, CA, USA). Strawberry GA 2-oxidase gene has been cloned earlier [40]. Full-length cDNA of *FaGA2ox *was obtained from Dr. Asaph Aharoni (Plant Research International, Wageningen, The Netherlands).

### Identification of putative GA signalling and target sequences

*Fragaria *sequences homologous to *Arabidopsis *GA biosynthetic, signalling and target genes were searched for in the Genbank nucleotide and EST databases by using the tBLASTn algorithm. Homologous strawberry sequences were further analysed by the BLASTx algorithm, and only sequences showing the highest sequence homology with the corresponding *Arabidopsis *genes were included in real-time RT-PCR analysis.

### Real-time RT-PCR

cDNAs for real-time reverse transcription PCR were synthesized from total RNA using a Superscript III RT kit (Invitrogen, Carlsbad, USA) and dT_18_VN anchor primers. LightCycler 480 SYBR Green I Master kit (Roche Diagnostics) was used to perform 15 μl real-time RT-PCR reactions in 384-well plates according to the manufacturer's instructions (Light Cycler 480 real-time PCR system, Roche Diagnostics). PCR primers with T_m _value of 60°C were used (Table 4), and the presence of single PCR product was verified by dissociation analysis. Relative quantification was performed by using *UBI14 *or *UBI14 *+ *Rubisco small subunit 1a *as control genes for axillary bud or runner samples, respectively.

**Table 4:** Real-time-PCR primers used in this study.

Gene	Forward primer 5' = > 3'	Reverse primer 5' = > 3'
*Rubisco small subunit 1a*	tggcctccagttggtttgaa	ccatttgttgcggagaagga
*Ubiquitin 11*	cagaccagcagaggcttatctt	ttctggatattgtagtctgctaggg
*GA3ox*	cctcacaatcatccaccaatcc	cgccgatgttgatcaccaa
*GA2ox*	caccatgcccagagcttca	aggccagaggtgttgttggat
*GID1b*	gtcccagttgacggtgtctt	attcacaaagccccattgag
*GID1c*	ttccatggtggaagctttgc	accgagacgacaacagcattg
*RGA*	tcagctggcttcggatactgtt	ttaccggctcgaaattcgg
*GAI*	cgattctcgacttcgctgac	atctgctggtggttctgcat
*SLY*	tggccgaggaggacgatta	ccctggtccttcaacgatcac
*SPY*	tgcggtgtcaaattgcatca	ggcaacactcaagatggattgc
*GAST1*	accctgttgaggctgatgga	ccgagacgataaacgacacctt
*XERICO*	ccacttgacttgtggccatgt	ctcctccacaggcattaaagga

T_m _value of the primers is 60 ± 1°C.

### Statistical analyses

Growth measurement data were subjected to one-way ANOVA when applicable. Comparisons of the means were done by Tukey's test. Statistical analyses were conducted using NCSS 2000 software.

## Authors' contributions

TH participated in the design of the study, carried out most of the experimental work and drafted the manuscript. TM analysed GA levels in the axillary buds. PE and OJ participated in the design and coordination of the study and helped to draft the manuscript. All authors read and approved the final manuscript.
